# Direct economic burden of hepatitis B virus related diseases: evidence from Shandong, China

**DOI:** 10.1186/1472-6963-13-37

**Published:** 2013-01-31

**Authors:** Jingjing Lu, Aiqiang Xu, Jian Wang, Li Zhang, Lizhi Song, Renpeng Li, Shunxiang Zhang, Guihua Zhuang, Mingshan Lu

**Affiliations:** 1 Center for Health Management and Policy in Shandong University, Jinan, Shandong, 250012, China; 2 Shandong Center for Disease Control and Prevention, Jinan, Shandong, 250014, China; 3 Shenzhen Center for Disease Control and Prevention, Shenzhen, 51800, China; 4 Department of Epidemiology and Biostatistics, Xi'an Jiaotong University College of Medicine, Xi’an, Shanxi, 710049, China; 5 Department of Economics, University of Calgary, Calgary, Alberta, T2N4T4, Canada

## Abstract

**Background:**

Although the expenses of liver cirrhosis are covered by a critical illness fund under the current health insurance program in China, the economic burden associated with hepatitis B virus (HBV) related diseases is not well addressed. In order to provide evidence to address the economic disease burden of HBV, we conducted a survey to investigate the direct economic burden of acute and chronic hepatitis B, cirrhosis and liver cancer caused by HBV-related disease.

**Methods:**

From April 2010 to November 2010, we conducted a survey of inpatients with HBV-related diseases and who were hospitalized for seven or more days in one of the seven tertiary and six secondary hospitals in Shandong, China. Patients were recorded consecutively within a three-to-five month time period from each sampled hospital; an in-person survey was conducted to collect demographic and socio-economic information, as well as direct medical and nonmedical expenses during the last month and last year prior to the current hospitalization. Direct medical costs included total outpatient, inpatient, and self-treatment expenditures; direct nonmedical costs included spending on nutritional supplements, transportation, and nursing. Direct medical costs during the current hospitalization were also obtained from the hospital financial database. The direct economic cost was calculated as the sum of direct medical and nonmedical costs. Our results call for the importance of implementing clinical guideline, improving system accountability, and helping secondary and smaller hospitals to improve efficiency. This has important policy implication for the on-going hospital reform in China.

**Results:**

Our data based on inpatients with HBV-related diseases suggested that the direct cost in US dollars for acute hepatitis B, severe hepatitis B, chronic hepatitis B, compensated cirrhosis, decompensated cirrhosis and primary liver cancer was $2954, $10834, $4552, $7400.28, $6936 and $10635, respectively. These costs ranged from 30.72% (for acute hepatitis B) to 297.85% (for primary liver cancer) of the average annual household income in our sample. Even for patients with health insurance, direct out-of-pocket cost of all HBV-related diseases except acute hepatitis B exceeded 40.00% of the patient’s disposable household income, making it a catastrophic expenditure for the household.

**Conclusion:**

Hepatitis B imposes considerable economic burden on a family. Our findings will help health policy makers’ understanding of the magnitude of the economic burden of HBV-related diseases in China. Evidence from our study also contributes to our understanding of potential benefits to society from allocating more resources to preventing and treating HBV infection, as well as increasing insurance coverage in China. These findings have important policy implications for health care reform efforts currently underway in China focusing on how to reduce the burden of catastrophic disease for its citizens.

## Background

According to the fourth national socio-epidemiological survey, the prevalence of hepatitis B surface antigen among Chinese aged less than 60 was 7.20% in 2006 [1]. At present, there is no cure for chronic hepatitis B (CHB) related disease and its complications. Clinicians mainly adopt antiviral treatment or immunomodulatory methods to improve liver function, and anti-fibrosis treatment to slow down the progression of the disease. The nature of lasting and recurring conditions from HBV infection compounded by the frequency of delayed consultation impose a heavy economic burden on patients with HBV and their families. It is therefore of great policy interest whether health insurance in China is adequate in terms of reducing the economic burden for those with chronic HBV. Although the direct economic burden of CHB in China has been estimated in a few studies [2-6], none of these studies estimated the direct out-of-pocket cost of all HBV-related diseases, including acute hepatitis B, severe hepatitis B, chronic hepatitis B, compensated cirrhosis, decompensated cirrhosis, and primary liver cancer.

In order to fill this gap, we conducted a survey and collected information on patients from thirteen representative hospitals in Shandong, China to investigate the direct costs of HBV-related diseases and their impact on households. This study aimed to provide evidence that would contribute to our understanding of the potential benefits to society of allocating more resources to preventing and treating HBV infection, as well as expanding insurance coverage for those with HBV-related diseases in China. This research has important policy implications for ongoing health care reform in China aimed at reducing the economic burden of catastrophic disease for its citizens.

## Methods

### Study population: the Shandong HBV-related inpatient economic burden survey

Between April and November 2010, we conducted a hospital survey of inpatients with HBV-related diseases in Shangdong province, China. We first used information from the Major Epidemic Network database to identify hospitals in all seventeen cities in Shandong which treated HBV-related patients in 2010. The Department of Health’s National Centre for Disease Control requires all hospitals in China to provide monthly reports of patients hospitalized for the treatment of HBV-related illness to the Major Epidemic Network. All 17 prefecture Centers for Disease Control and Prevention in Shandong province have been required to regularly report number of cases with hepatitis B virus related disease through the National Notifiable Disease Surveillance System (NNDSS). This information in the NNDSS was used to partly guide our data sampling. First, out of the prefectures with the largest reported number of cases in NNDSS, we chose three prefectures – Qingdao, Jinan, and Liaochang – as representative prefectures of the province. Geographically, these prefectures are located in the east, central, and west part of the province. Each represents different level of economic development: west being the most, central next, and east the least developed. Then, within each chosen prefecture, two tertiary or secondary hospitals were randomly chosen. Finally, our interviewers went sent to these selected hospitals to conduct the survey in September 2010.

**Figure 1:**
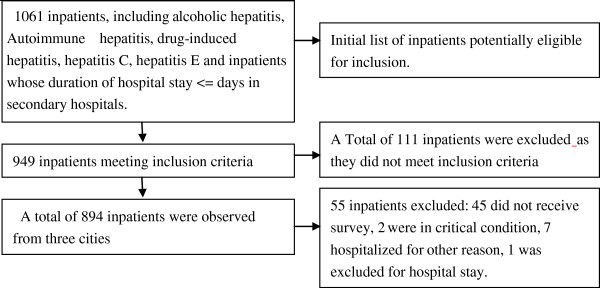
A summary of sampling participants for interview.

In our survey, ICD-10 was used to identify eligible cases (Table 1). Inpatients were recorded consecutively within a time period of three to five months from each of the thirteen sampled hospitals. Patient sampling was ended when the number of qualified observations reached 400 in each city, except in Liaocheng where the patients totaled less than 200. Patients eligible to be included in the survey included those who were admitted into a hospital for seven or more days due to HBV-related disease and its complications, including acute hepatitis B, severe hepatitis B, chronic hepatitis B, compensated cirrhosis, decompensated cirrhosis, and primary liver cancer. The diagnostic standard in this paper for severe hepatitis B is: Prothrombin activity below 40% and serum bilirubin more than 10 times normal. We excluded patients with toxic, drug induced and immune hepatic disease, viral hepatitis caused by a secondary liver virus, as well as patients with HBV-related diseases whose hospitalization was for another reason. This resulted in a total of 949 observations in three cities (Figure 1).

**Table 1:** ICD-10 codes of HBV-related diseases

**Classification**	**ICD 10 code**	**ICD 10 title**
Acute hepatitis B	B16.902	Acute hepatitis B without icteric
	B16.901	Acute hepatitis B with icteric
Severe hepatitis B	B16.906	Severe hepatitis B
	B16.908	Acute Severe hepatitis B
	B16.909	subacute severe hepatitis B
	B18.102	Chronic severe hepatitis B
Compensated and decompensated cirrhosis	K74.601	cirrhosis of liver
Primary liver cancer	C22.001	Hepatocellular carcinoma

Interviews were conducted by 10 trained professional investigators to collect information on both outpatient and inpatient expenses during the 12 months prior to the current hospitalization. The interviewers were six postgraduate students of the Centre for Health Management and Policy at Shandong University whose major was social medicine and health management, as well as four staff members who were trained in public health with a specialty in epidemiology at the Shandong Provincial Centre for Disease Control and Prevention. Investigators explained the purposes and confidentiality of the survey, and then invited patients to participate in the survey. Respondents could choose not to participate; 894 agreed to be interviewed. Ethics approval of this study was obtained from the Shandong Provincial Centre for Disease Control and Prevention. After patients were discharged from the hospital, inpatient expenses for the most recent hospitalization were obtained from the hospital financial database.

### Key variables

In the survey, the following information was collected: hospital ID, demographic information and socioeconomic status (including age, gender, education, occupation, family size, and individual and household monthly income), diagnosis (ICD-10), and health insurance status. ICD-10 is collected from the hospital nursing stating where patients are diagnosed when admission. Therefore the interviewers would have had that information ahead of time. Health insurance was categorized as insured or uninsured. Insured was defined as someone who was covered by any of the following types of health insurance programs: public insurance provided to civil servants, the basic health insurance scheme for urban employees (BHISUE) or for urban residents (BHISUR), the New Rural Cooperative Medical Scheme (NRCMS), or private insurance.

The patients were interviewed during hospitalization about their expenditures of previous hospital stays within the last 12 months. The expenditures of their current hospital stays were extracted directly from the hospital financial database right after their discharge. Direct medical costs included total outpatient and inpatient expenditures (i.e., hospitalization expenditures, nursing, prescription drugs, examination fees, etc.), and total self-treatment expenditures. Direct total costs for each patient were divided by the number of admissions if the patient had two or more hospital admissions. Direct nonmedical costs included expenditures on nutrition supplements, transportation, and patient accompaniers’ costs as a result of the disease. Finally, information on inpatient expenditures for the current hospitalization was obtained from the hospital financial database. All costs are expressed in United States dollars using the June 30, 2010 exchange rate of 6.7909 RMB.

### Statistical analysis

Descriptive statistics were employed to illustrate the characteristics of both insured and uninsured patients in the study population; the characteristics included sociodemographic characteristics, out-patient and inpatient expenses, direct and in-direct costs, and health insurance status. Frequencies of variables in the survey were not weighted because sampling weights were not available. A parallel and double entry method was used to input data using Epidata 3.0; direct medical and nonmedical costs were calculated. Stata 7.0 was used for the statistical analysis in this study. As the costs were non-normally distributed, the Kruskal-Wallis test was used to test for statistically significant differences.

## Results

### Descriptive results

A descriptive analysis of both insured and uninsured HBV-related patients is provided in Table 2. Among the 894 HBV-related patients included in our survey, 347 cases were from Jinan, 374 cases from Qingdao and 173 cases from Liaocheng. There were 849 patients (94.97%) with insurance; 45 (5.03%) without insurance. Most patients (86.35%) had one of three conditions: chronic hepatitis B, compensated cirrhosis or decompensated cirrhosis. Mean age was 45.80 years and over two-thirds were male (641 or 71.70%). The insured group was older than the uninsured group (46.37 vs. 34.89). Most had obtained a high school education or above; only 19.69% were uneducated or only attended primary school. Household monthly income was $438 for the overall sample; $439 for the insured and $411 for the uninsured group. Individual monthly income was $190 for the overall sample; $190 for the insured and $191 for the uninsured.

**Table 2:** Characteristics of all HBV-related diseases for insured and uninsured inpatients in Shandong province

**Characteristics**	**All inpatients**	**Uninsured (%)**	**Insured (%)**
	**N = 894**	**N = 45**	**N = 849**
Location			
Jinan	347	5.19	94.81
Qingdao	374	5.89	94.11
Liaocheng	173	2.89	97.11
Condition			
Acute hepatitis B	29	4 (13.79%)	25 (86.21%)
Severe hepatitis B	28	0 (0.00%)	28 (100.00%)
Chronic hepatitis B	449	33 (7.35%)	416 (92.65%)
Compensated cirrhosis	121	1 (0.83%)	120 (99.17%)
Decompensated cirrhosis	202	6 (2.97%)	196 (97.03%)
Primary liver cancer	65	1 (1.54%)	64 (98.46%)
Male	71.7%	80	71.26
Age (sd)	45.8 (13.88)	34.89 (12.04)	46.37 (13.73)
Education			
Uneducated and primary school	19.69	8.89	20.26
High school	60.74	57.78	60.90
College	18.57	28.89	18.02
Graduate	1.01	4.44	0.82
household monthly income (sd)	438 (464)	411 (325)	439 (470)
Patient monthly income (sd)	190 (201)	191 (220 )	190 (201)

*Notes:

1. Monetary values reported in US dollars. 1 US dollar = 6.7909 RMB (exchange rate on Jun 30th, 2010).

2. Standard deviation is reported in the parentheses.

### Direct cost

Direct total costs for each patient were divided by the number of admissions if the patient had two or more hospital admissions. As shown in Table 3, the direct costs for acute hepatitis B, severe hepatitis B, chronic hepatitis B, compensated cirrhosis, decompensated cirrhosis, and primary liver cancer were $2954, $10834, $4552, $7400, $6937, and $10636 respectively. Direct costs for inpatients who were insured were higher than for the uninsured. Proportion of direct medical cost (%) is consistently high, accounting for 91-97% in direct cost. For insured patients, there was a significant difference in the direct costs among the HBV-related diseases with primary liver cancer costing much more.

**Table 3:** Inpatient direct costs of all HBV-related diseases (dollar/per admission)

**Classification**	**Direct cost**		
	**Insured**	**Uninsured**	**Overall mean**
acute hepatitis B	3098	2056	2954
severe hepatitis B	10834.	-	10834
chronic hepatitis B	4689	2825	4552
compensated cirrhosis	7451	1311	7400
decompensated cirrhosis	7046	3359	6937
primary liver cancer	10769	2099	10636
x^2^	**127.87**	**3.66**	**135.47**
*P*	**0.0001**	**0.4538**	**0.0001**

The direct medical cost of acute hepatitis B, severe hepatitis B, chronic hepatitis B, compensated cirrhosis, decompensated cirrhosis, and primary liver cancer was $2854, $10114, $4136, $6781, $6400, and $9891, respectively. As shown in Table 4, outpatient expenditures were highest for primary liver cancer ($1476), followed by decompensated cirrhosis and compensated cirrhosis at $958 and $951, respectively. Hospitalization stay expenditures were highest for severe hepatitis B and primary liver cancer, $9367 and $8209 respectively, followed by compensated cirrhosis and decompensated cirrhosis, $5762 and $5355, respectively. As for self-treatment expenditures, primary liver cancer and severe hepatitis B were the highest, estimated to be $206 and $191, respectively. Uninsured patients reported almost no self-treatment expenses. For insured patients, direct medical costs differed significantly among the six HBV-related conditions, with the highest spending being for severe hepatitis B ($10114. Clearly this disease has a major impact in terms of healthcare costs.

**Table 4:** Direct medical cost of all HBV-related diseases (dollar/per annual average admission)

**Classification**	**Outpatient expenditure**	**Hospitalization stay expenditure**	**Self-treatment expenditure**	**Total**
Acute hepatitis	109	2741	4	2854
B **(median)**	**(60)**	**(2332)**	**(0)**	**(2363)**
Severe hepatitis	557	9367	191	10114
B **(median)**	**(265)**	**(9395)**	**(0)**	**(9426)**
Chronic hepatitis	615	3508	13	4136
B **(median)**	**(133)**	**(2215)**	**(0)**	**(2745)**
Compensated	951	5762	67	6781
cirrhosis **(median)**	**(996)**	**(3152)**	**(0)**	**(3811)**
Decompensated	958	5355	88	6400
cirrhosis **(median)**	**(305)**	**(3754)**	**(0)**	**(4541)**
Primary liver	1476	8209	206	9891
cancer **(median)**	**(449)**	**(5941)**	**(0)**	**(7260)**
x^2^	41.87	126.01	24.76	131.63
*P*	0.0001	0.0001	0.0002	0.0001

The study found that the direct nonmedical cost of acute hepatitis B, severe hepatitis B, chronic hepatitis B, compensated cirrhosis, decompensated cirrhosis and primary liver cancer, was $100, $719, $416, $619, $536 and $745. Transportation costs were $83, $537, $113,$235, $230 and $494, respectively, and were higher for insured versus uninsured patients. Nutrition expenses were $16, $170, $302, 383, $303 and $251, respectively. There were no statistically significant differences in the direct nonmedical cost of HBV-related diseases among those who did not have insurance. However, direct nonmedical costs of those who had insurance were highest for patient’s primary liver cancer.

### Impact of disease burden on a household

The direct economic burden of HBV-related diseases and their impact on a household were illustrated in Table 5. The direct cost of HBV-related diseases as a proportion of annual family income ranged from 30.72% for those with acute Hepatitis B to 297.85% for those with primary liver cancer. Even after reimbursement, the direct cost of patients who experienced severe hepatitis B, chronic hepatitis B, compensated cirrhosis, decompensated cirrhosis, and primary liver cancer exceeded 40.00% of disposal household income. The economic burden was significantly different among the six diseases for insured patients.

**Table 5:** The impact of disease burden on a household (dollar/per annual average admission)

**Classification**	**Direct cost**				**Annual household income**			**Percent of direct cost in annual household income (%)**			
	**Insured (before reimbursement)**	**Insured (after reimbursement)**	**Uninsured**	**Overall**	**Insured**	**Uninsured**	**Overall**	**Insured (before reimbursement)**	**Insured (after reimbursement)**	**Uninsured**	**Overall**
acute hepatitis B **(median)**	3098	1549	2056	2954	10171	6141	9615	30.46	15.23	33.48	30.72
	**(2779)**	**(1389)**	**(1469)**	**(2464)**	**(5301)**	**(5213)**	**(5301)**	**(52.42)**	**(26.21)**	**(28.18)**	**(46.49)**
severe hepatitis B **(median)**	10834	5417	—	10834	3941	—	3941	274.89	137.45	—	274.89
	**(9699)**	**(4849)**		**(9697)**	**(3534)**		**(3534)**	**(274.42)**	**(137.20)**		**(174.39)**
chronic hepatitis B **(median)**	4689	2345	2825	4552	5824	5191	5778	80.52	40.26	54.42	78.79
	**(2978)**	**(1489)**	**(2673)**	**(2919)**	**(4418)**	**(4241)**	**(4418)**	**(67.41)**	**(33.70)**	**(63.02)**	**(66.07)**
compensated cirrhosis **(median)**	7451.	3726	1311	7400	5297	3534	5282	140.67	70.34	37.09	140.10
	**(4116)**	**(2058)**	**(0.00)**	**(4074)**	**(3711)**	**(3534)**	**(3711)**	**(110.93)**	**(55.46)**	**(37.09)**	**(109.77)**
decompensated cirrhosis **(median)**	7046	3523	3359	6937	4187	3475	4166	168.30	84.15	96.65	166.52
	**(4980)**	**(2490)**	**(3423)**	**(4946)**	**(3534)**	**(2474)**	**(3534)**	**(140.91)**	**(70.45)**	**(138.37)**	**(139.95)**
primary liver cancer **(median)**	10769	5385	2099	10636	3602	1590	3571	298.99	149.50	131.99	297.85
	**(7977)**	**(3988)**	**(2099)**	**(7884)**	**(2827)**	**(1590)**	**(2827)**	**(282.15)**	**(141.06)**	**(131.99)**	**(278.86)**
*x*^2^	127.87	**127.00**	**3.66**	**135.47**							
*P*	0.0001	**0.0001**	**0.45**	**0.0001**							

### Re-analyses after excluding the 45 uninsured patients

We re-ran the analyses after excluding the 45 uninsured patients since some of the conditions were either not represented (severe hepatitis B) or have very few patients (acute hepatitis B, compensated cirrhosis, primary liver cancer) among uninsured patients (Table 2). In this analysis, costs were stratified by level of care since costs may differ between tertiary and secondary hospitals.

As shown in Table 6, the direct cost for acute hepatitis B, severe hepatitis B, chronic hepatitis B, compensated cirrhosis, decompensated cirrhosis, and primary liver cancer in tertiary hospitals was $3135, $10834, $4380, $6022, $7107, and $10213 respectively. The direct cost for acute hepatitis B, chronic hepatitis B, compensated cirrhosis, decompensated cirrhosis, and primary liver cancer in secondary hospitals was $1388, $5588, $19919, $6565, and $13268, respectively. The direct costs for acute hepatitis B were significantly higher in tertiary hospitals compared to secondary hospitals (*x*^2^ = 4.31, *P* = 0.0378), whereas the opposite was true for those with chronic hepatitis B (*x*^2^ = 12.12, *P* = 0.0005).

**Table 6:** The direct cost of all HBV-related diseases stratified by hospitals (dollar/per average annual admission)

**Classification**	**Tertiary hospitals**		**Secondary hospitals**		**Total**		**x**^**2**^	***P***
	**NO.**	**cost**	**NO.**	**cost**	**NO.**	**cost**		
Acute hepatitis B **(median)**	26	3135	3	1388	**29**	2954	**4.31**	**0.0378**
		**(2830)**		**(1598)**		**(2464)**		
Severe hepatitis B **(median)**	28	10834	0	—	**28**	10834	**—**	**—**
		**(9697)**			**449**	**(9697)**		
Chronic hepatitis B **(median)**	385	4380	64	5588		4552	**12.12**	**0.0005**
		**(3012)**		**(2131)**		**(2919)**		
Compensated cirrhosis **(median)**	109	6022	12	19919	**121**	7400	**0.08**	**0.7747**
		**(3909)**		**(4586)**		**(4074)**		
Decompensated cirrhosis **(median)**	170	7107	32	6565	**202**	6937	**1.99**	**0.1583**
		**(5042)**		**(4177)**		**(4946)**		
Primary liver cancer **(median)**	56	10213	9	13268	**65**	10636	**1.39**	**0.2390**
		**(7595)**		**(11223)**		**(7884)**		
x^2^		115.20		21.99		**135.47**		
*P*		0.00		0.00		**0.0001**		

As shown in Table 6, median cost of patients with acute hepatitis B treated in secondary hospitals is significantly lower than those treated in tertiary hospitals (1388 vs. 3135, p < 0.04). ). It should be noted, however, that the sample size of patients with acute hepatitis B treated in secondary hospitals is quite low (3 only) and the results on cost comparison may not be meaningful. For patients with chronic hepatitis B however, 64 were treated in secondary hospitals and 385 in tertiary hospitals. The median cost of those treated in secondary hospitals is significantly higher (5588 vs. 4380, p < 0.00).

## Discussion

Even with health insurance coverage, the direct costs of HBV-related diseases, except acute hepatitis B, exceeded 40.00% of household annual income in China, suggesting that HBV-related diseases should be placed categorized as catastrophic [7,8]. Due to the large amount of economic resources consumed, our results indicate that HBV-related diseases imposed a substantial economic burden on patients and their families in China [9,10].

Our findings demonstrate that the direct economic burden of HBV infection increases as the disease progresses, with severe hepatitis B and primary liver cancer incurring expenses close to three times annual household income. By far the largest portion of direct costs, over 90 per cent, were medical in nature, consistent with Li et al.’s findings that 93.07% of direct costs were medical [6]. Much of the direct medical costs (84.86%) were due to the costs of hospitalization, mainly due to drugs and examination fees which accounted for 77.49% of hospitalization costs.

Our study has several limitations. First, only tertiary and secondary hospitals were included in our survey. No township hospitals were sampled because the number of patients with HBV-related diseases in these hospitals was very small. Second, other than the direct medical cost for the current hospitalization, which was obtained from hospital financial data, all other outpatient and inpatient costs were self-reported and may be subject to recall error. Third, our data only allowed us to assess the direct inpatient cost of HBV-related diseases. We did not have data to estimate the direct outpatient cost of HBV-related diseases, nor were we able to evaluate the indirect cost of HBV-related diseases such as loss of productivity and income as a result of illness days and hospitalization, or loss of income for family members who were caregivers. In other words, the actual total economic burden of HBV-related diseases could be either higher or lower than our estimates. On the other hand, however, our survey only included patients who were hospitalized for seven or more days. These are likely patients with higher severity of illness which would cause upward bias in our estimations. Finally, our study does not capture the full burden of disease because it does not survey individuals infected with hepatitis B who were not hospitalized during the sampling period. Future research should address the important issue of estimating the overall economic burden of HBV-related diseases in China.

Our data indicated that median cost of chronic hepatitis B patients in secondary hospitals was significantly higher than those in tertiary hospitals. Considering the fact that patients with more severe illnesses and thus more costly patients would have been more likely to seek treatment at tertiary hospital, this result is alarming. This might be an indication of higher level of inefficiencies among secondary hospitals, as compared with tertiary hospitals. Clinical guideline is still under development in China, and hospital inefficiency has been a huge concern in the current hospital system. While tertiary hospitals attract the best resources, secondary hospitals in China are in a much more disadvantaged position and as a result, quality of care and efficiency could be much bigger issues. Our results call for the importance of implementing clinical guideline, improving system accountability, and helping secondary and smaller hospitals to improve efficiency. This has important policy implication for the on-going hospital reform in China.

## Conclusion

Hepatitis B imposes considerable economic burden on a family. Our findings will help health policy makers’ understanding of the magnitude of the economic burden of HBV-related diseases in China. Evidence from our study also contributes to our understanding of the potential benefits to society of allocating more resources to preventing and treating HBV infection, as well as increasing insurance coverage in China. For instance, patients with severe hepatitis B or primary liver cancer suffered a high economic burden even after insurance reimbursement, spending 137.45% and 149.50%, respectively, of their household annual income. These findings have important policy implications for health care reform efforts currently underway in China focusing on how to reduce the burden of catastrophic disease for its citizens. In addition, overtreatment and over-medication are among the reasons for high personal costs in private facilities, given that government funding to public providers is insufficient. It is important to note that funds currently used for the unnecessary treatment of acute hepatitis B and for nutrition supplements could be redirected to programs designed to reduce the economic burden of HBV-related diseases.

## Consent

Written informed consent was obtained from the patient for publication of this report and any accompanying images.

## Competing interests

The authors declare that they have no competing interests.

## Authors’ contributions

JL, XA and JW conceptualized and supervised the study, contributed to the study design, made substantial contributions to the acquisition and qualityassurance of the data, and analyzed the data. LZ, LS, RL, SZ, and GZ contributed to the study design, survey conduction and supervision, as well as interpretation and writing of the manuscript. ML contributed to the statistical analysis, interpretation, writing and finalizing of the manuscript. All authors read and approved the final manuscript.

## Pre-publication history

The pre-publication history for this paper can be accessed here:

http://www.biomedcentral.com/1472-6963/13/37/prepub
